# On defining quantifying and measuring the SNARC effect

**DOI:** 10.3389/fpsyg.2013.00302

**Published:** 2013-05-30

**Authors:** Joseph Tzelgov, Bar Zohar-Shai, Hans-Christoph Nuerk

**Affiliations:** ^1^Department of Psychology, Ben-Gurion University of the NegevBeer-Sheva, Israel; ^2^Department of Brain and Cognitive Sciences, Zlotowski Center for Neuroscience, Ben-Gurion University of the NegevBeer Sheva, Israel; ^3^Department of Psychology, Eberhard Karls UniversityTübingen, Germany; ^4^Knowledge Media Research Center, IWM-KMRCTübingen, Germany

About twenty years ago, Dehaene et al. (1993) asked participants to perform a parity task twice, each time with a different mapping of response hand to parity value. The participants responded with a key-press and latency served as the dependent variable. The two mappings are depicted in Panel **(A)** of Figure 1. The yellow background cells correspond to one mapping of parity to the possible responses: the odd (red) numbers are responded to by a left key-press and the even (blue) numbers are responded to by a right key-press. The green background cells correspond to the alternative mapping: the even (blue) numbers are responded to by the left key-press and the odd (red) numbers are responded to by a right key-press.

**Figure 1:**
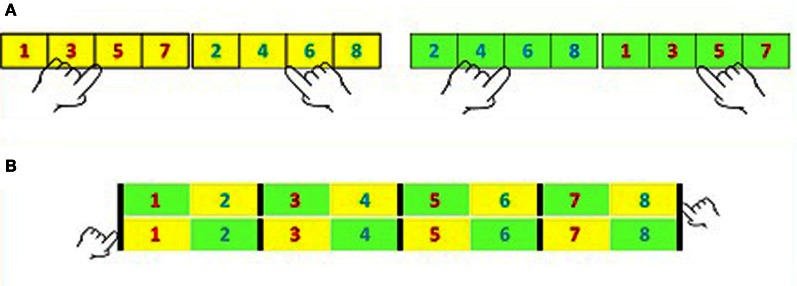
**(A)** The two mappings of parity. **(B)** The post-experimental classification of stimuli.

## Defining the SNARC effect

Panel (**B)** corresponds to a post experimental classification of the experimental conditions. In this panel, Hands (H) correspond to rows, and color of digits to Parity (P). Magnitude (M) is defined in terms of the two adjacent numbers, one odd and one even. When the numbers 1–8 are used (see Figure 1), this results in four categories of magnitude as indicated by the black lines in the panel. Note that in Panel (**B**), odd and even numbers come from different mappings and thus by defining Magnitude as such, we average over the mapping.

The three factors H, M, and P are of main interest in Dehaene et al.'s (1993) experiments and in the studies modeled after them. In particular, the interaction between Hand and the linear contrast of Magnitude, within the three-way Hand by Magnitude by Parity design as defined by Dehaene and colleagues as the Space-Number Association of Response Codes (SNARC) effect. Let us have a deeper look at the components of the Sum of Squares of Effects^1^ (SSEff) of the various factors manipulated in this design.

(1) SSEff=SSH+SSP+SSM+SSHP+ SSHM+SSMP+SSHMP

where SSH, SSP, and SSM refer to the Sums of Squares due to Hand, Parity, and Magnitude, respectively, and the remaining components refer to their interactions. Note that the SNARC effect is defined as the interaction between Hand and the linear component of Magnitude, which is part of the SSHM; the Sum of Squares due to Hand-Magnitude interaction. Another interesting component of the SSEff is the Hand-Parity interaction. This interaction defines the Markedness Association of Response Codes (MARC) effect as analyzed by Nuerk et al. (2004). This effect refers to faster responses in the parity task when even numbers are responded to with the right hand and odd numbers are responded to with the left hand (see the yellow cells in Figure 1) than in the opposite mapping (see the green cells in Figure 1).

## Quantifying the SNARC effect

The definition of the SNARC effect in terms of the interaction between hand and the linear contrast of magnitude *per se*, allows testing the existence (i.e., significance) of the SNARC effect. However, it is not an estimation for the size of the effect.

Such estimate is usually provided by referring to Panel (**B**), which we just described as an arrangement consistent with a three-way description of SNARC experiments, in terms of a two-way factorial design with Hands corresponding to the rows of the table in Panel **(B)** and Numbers to it columns. This allows estimating the squared correlation (*r*^2^_*Nd(RT)*_) between Numbers and dRT—the difference between the latencies in responding to each number with the right vs. the left hand. As shown by Pinhas et al. (2012), *r*^2^_*Nd(RT)*_ can be estimated within the analysis of variance (ANOVA) design as *r*^2^_HN_ which equals

(2) $$r_{\text{HN}}^{2}=\frac{\text{SSL}(\text{HN})}{\text{SSHN}}$$

where SSL(HN) refers to the linear component of the HN interaction and SSHN refers to the interaction between Hand and Numbers.

Of course referring to the same data in a different way does not change the SSEff, which can be also written in terms of the components of a two-way description of the design in terms of Hand and Number as:

(3) SSEff=SSH+SSN+SSHN.

It can be shown that:

(4) SSN=SSM+SSP+SSMP

and what is more important from the perspective of the present discussion, it can also be shown that:

(5) SSHN=SSHM+SSHP+SSHMP

This means that, to the variability due to the Hand-Number interaction, Hand contributes not just to the interaction between Hand and Magnitude but also to the interaction between Hand and Parity and the triple interaction among Hand, Magnitude, and Parity.

It follows that if the SNARC effect is defined in terms of the linear component of the Hand and Magnitude interaction but is most frequently quantified by (*r*^2^_*Nd(RT)*_) due to the linear component of Hand-Number interaction, there is an inconsistency between the definition of the SNARC effect, on the one hand, and the quantification of its effect size, on the other. In particular, the definition refers to the linear relation between the Magnitude (average of two adjacent numbers presented) and dRT, while the quantification refers to the linear relation of Number and dRT.

To keep consistency, the SNARC effect should be quantified as *r*^2^_*HM*_, the squared correlation between Hand and Magnitude. Given Pinhas et al.'s (2012) analysis, this can be done easily within the ANOVA framework as:

(6) $$r_{\text{HM}}^{2}=\frac{\text{SSL}(\text{HM})}{\text{SSHM}}$$

where SSL (HM) refers to the linear component of the HM interaction and the SSHM refers to the interaction between Hand and Magnitude. To the best of our knowledge, until now *r*^2^_*HM*_ has never been reported as a quantification of the SNARC effect. Yet, we believe that using the three way H × M × P design and quantifying the SNARC effect by *r*^2^_*HM*_ is the preferred solution. First, it allows sticking with the original definition (Dehaene et al., 1993). Second, by being uncontaminated by the HP interaction, which is the source of the MARC effect, it allows a better estimation of the SNARC effect. Note that this implies estimating the relation to the mental number line as the linear component of the HM interaction [see Equation (6)]. By contrast, two of the components of the HN interaction (SSHP and SSHPM) are not part of the linear relation between the mental number line and the magnitudes corresponding to the number presented. Note that the HP interaction, which is realized as the MARC effect, underestimates the latencies when there is Parity-Hand correspondence, and overestimates the latencies in the absence of such correspondence. We expect these changes in estimates, which are irrelevant to the linear relation between number magnitude and dRT, to decrease the SNARC effect whenever there is an interaction between Hand and Parity. We predict this decrease to happen in particular in languages in which morphology contributes to markedness (Clark, 1969). German and Hebrew are two examples in which “odd” is expressed as the explicit negation of “even” (i.e., “un-even”).

## Reporting the SNARC effect

In the original study of Dehaene et al. (1993), the SNARC effect was reported as the proportion of explained variance (*r*^2^) providing a measure of the effect size of the SNARC effect. In addition, the slope of the regression in terms of ms/number (millisecond per number) was provided. Reporting the slope is very helpful by providing an intuitive interpretation of movement along the number line. Fias et al. (1996) applied the method proposed by Lorch and Myers (1990) for mixed model regression to the analysis of the dRT number relation. This method allows testing the significance of the linear relation between numbers and dRT. Consequently, the (unstandardized) slope of this regression has become the most frequently reported measure of the SNARC effect. However, it does not provide an estimate of the correlation between the dRT and number magnitude. Nevertheless it has incorrectly been interpreted as the effect size of the SNARC effect, which it clearly is not! It should be clear that, as pointed out by Lorch and Myers, the semi-partial squared correlation between dRT and Numbers could be used as a measure of the size of the SNARC effect but in most cases it is not reported.

## Conclusions

In closing we would like to emphasize several points. First, we do believe that the SNARC effect is a useful marker of the Number-Space association. We do think that we should focus on numbers as mathematical entities corresponding to specific magnitudes. Therefore, and in order to avoid effects of other features of numbers (and in particular, parity), the SNARC effect should be defined in terms of the relation of number magnitude and dRT rather than to numbers (*per se*) and dRT because other numerical attributes may interfere with or facilitate the SNARC effect if they are not considered. As explained above, this should be done also for the sake of consistency. In addition, it is important to report the size of the SNARC effect and this should not be done in terms of the (unstandardized) slope of the regression function. Such a slope is helpful by providing an intuitive grasp of the SNARC effect but it is not a measure of effect size. Finally, we recommend analyzing the SNARC effect by applying an ANOVA approach as proposed by Pinhas et al. (2012), which allows doing so in a simple and efficient way.

## Footnotes

^1^We define the SSEff as the sum of squares of all effects tested in this design. It does not include the errors terms (the interaction of each effect with the Participants factor).
